# Why Is Very High Cholesterol Content Beneficial for the Eye Lens but Negative for Other Organs?

**DOI:** 10.3390/nu11051083

**Published:** 2019-05-15

**Authors:** Justyna Widomska, Witold K. Subczynski

**Affiliations:** 1 Department of Biophysics, Medical University of Lublin, Jaczewskiego 4, 20-090 Lublin, Poland; 2 Department of Biophysics, Medical College of Wisconsin, 8701 Watertown Plank Road, Milwaukee, WI 53226, USA; subczyn@mcw.edu

**Keywords:** cholesterol, phospholipid membrane, eye lens, arterial cells, cataract, atherosclerosis

## Abstract

The plasma membranes of the human lens fiber cell are overloaded with cholesterol that not only saturates the phospholipid bilayer of these membranes but also leads to the formation of pure cholesterol bilayer domains. Cholesterol level increases with age, and for older persons, it exceeds the cholesterol solubility threshold, leading to the formation of cholesterol crystals. All these changes occur in the normal lens without too much compromise to lens transparency. If the cholesterol content in the cell membranes of other organs increases to extent where cholesterol crystals forma, a pathological condition begins. In arterial cells, minute cholesterol crystals activate inflammasomes, induce inflammation, and cause atherosclerosis development. In this review, we will indicate possible factors that distinguish between beneficial and negative cholesterol action, limiting cholesterol actions to those performed through cholesterol in cell membranes and by cholesterol crystals.

## 1. Introduction

The plasma membrane of the human eye lens fiber cell is unique in its enormous cholesterol content, which increases, over time, to a level that is incomparable with other tissue/organ cells [1,2]. At this high content, cholesterol not only saturates the lipid bilayer portion of fiber cell membranes but also induces formation of pure cholesterol bilayer domains (CBDs) within this bilayer. At old age, when the cholesterol content exceeds the cholesterol solubility threshold, the excess cholesterol forms cholesterol crystals, presumably outside the membrane [2]. In addition to the increased cholesterol content that occurrs with age, the phospholipid composition of fiber cells changes drastically [3,4,5]. None of these age-related changes affect the function of the lens, and in healthy people, the lens can remain transparent until old age. In the plasma membranes of cells of other human tissues and organs, the typical cholesterol/phospholipid molar ratio is between 0.1 and 0.5 [6], and does not change significantly with age. When the cholesterol content in these membranes increases above the cholesterol solubility threshold and cholesterol crystals start to form, this is a sign of a pathological condition. [7]. For instance, it was shown that the deposition of minute cholesterol crystals in arterial cells initiates and promotes atherosclerosis [8,9,10].

It should be recognized that the lens is an avascular structure, and not all the nutrients available in blood can easily penetrate the lens. It is also true that dietary cholesterol does not influence cholesterol content in the eye lens [11]. The opposite situation exists in cells of other human tissues and organs, where a high cholesterol level in blood and oxidative stress are major factors leading to the development of atherosclerosis [12]. In this review, we discuss why high cholesterol content is beneficial, and even necessary, to maintain the fiber cell plasma membrane, fiber cell itself, and whole lens homeostasis. We also discuss why the formation of CBDs and cholesterol crystals in other human tissues and organs is a sign of a pathological condition [13,14] but is not harmful to the eye lens [2].

## 2. Human Lens Cholesterol

### 2.1. Cholesterol Synthesis

**Figure 1:**
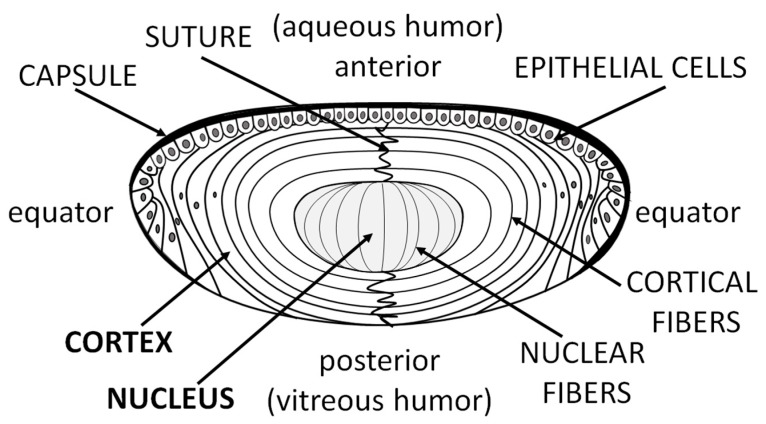
Diagram of the eye lens, showing the location of the lens cortex, nucleus, epithelial cells, and layers composed of hundreds of fiber cells. Fiber cells are extremely long, stretching from anterior to posterior.

An avascular lens derives nutrients from the aqueous humor. The anterior and equatorial lens epithelium form a barrier between the aqueous humor and the fiber cells of the lens, which form the bulk of the lens. Epithelial cells, which occupy only a minor part of the lens (Figure 1), form the most metabolically active part of the lens. Fiber cells, which form the lens body, possess significantly lower metabolic activity.

As compared with lenticular proteins, much less is known about lipid metabolism and synthesis in the lens, especially regarding cholesterol. However, similar to phospholipids [15], the cholesterol level should be independent of diet. Zelenka reported that the lens can synthesize and remodel its own lipids [16]. Similarly, cholesterol should be synthesized in the lens [17,18]. The biosynthesis of cholesterol by the lens was confirmed by Cenedella [11] with the finding that during the first two weeks of life, the lens of rat can obtain most of its cholesterol through de novo synthesis. It is well known that cholesterol content increases with age, but no correlation has been found between the rate of cholesterol synthesis and the level of cholesterol increase during aging [19].

**Figure 2:**
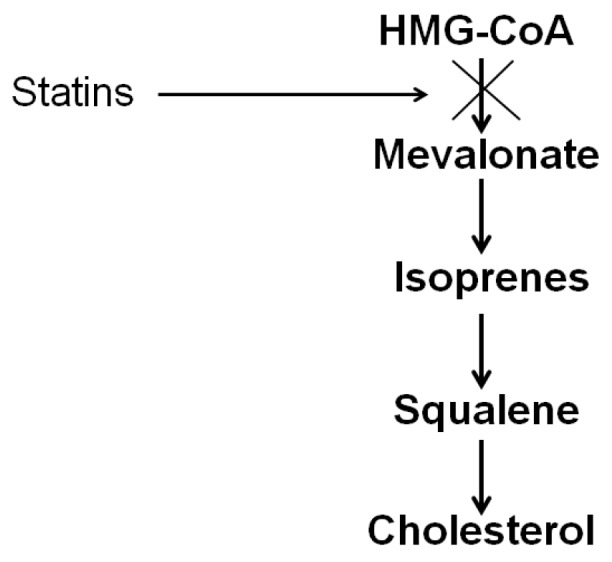
Main stages in the cholesterol biosynthesis pathway.

The cholesterol synthesis process can be simply described as being composed of four main stages (Figure 2). Similar to the synthesis of long-chain fatty acids, the synthesis of cholesterol begins with the two-carbon acetate group of acetyl-CoA. Initially, three acetate units condense to form the six-carbon intermediate mevalonate. In the second stage, mevalonate is phosphorylated to activated isoprene, namely, isopentyl pyrophosphate. In the third stage, the condensation of six activated isoprene results in the formation of squalene. Finally, the fourth stage involves conversion of the linear squalene molecule to the four-ringed steroid. The commonly prescribed cholesterol-lowering medications, statins, impair endogenous cholesterol production by inhibiting HMG-CoA reductase, the enzyme that catalyzes conversion of HMG-CoA to mevalonate (Figure 2). Vries at al. observed that the cholesterol content of the rat lens is lowered by simvastatin but not by pravastatin [20]. They concluded that accumulation of the lenticular cholesterol with age is dependent on the in situ de novo synthesis.

The aqueous humor provides nutrition and removes metabolic waste from avascular tissues such as the lens. As a result of this, the maintenance of lens transparency is strongly dependent on the diffusion of nutrients to and from that fluid. However, it is still unclear if the lens obtains cholesterol from lipoproteins, as do other tissues. Only a trace amount of cholesterol was found in the aqueous humor (~1 μg/mL) [21] compared with plasma (~200 mg/dL) [22]. Cholesterol found in the human aqueous humor was present solely in the form of high-density lipoprotein (HDL), the concentration of which is also negligible (~4 μg/mL) [21]. Thus, HDL in the aqueous humor might be only a minor source of lenticular cholesterol, if it is a source at all.

### 2.2. Changes with Age and Cataract

**Figure 3:**
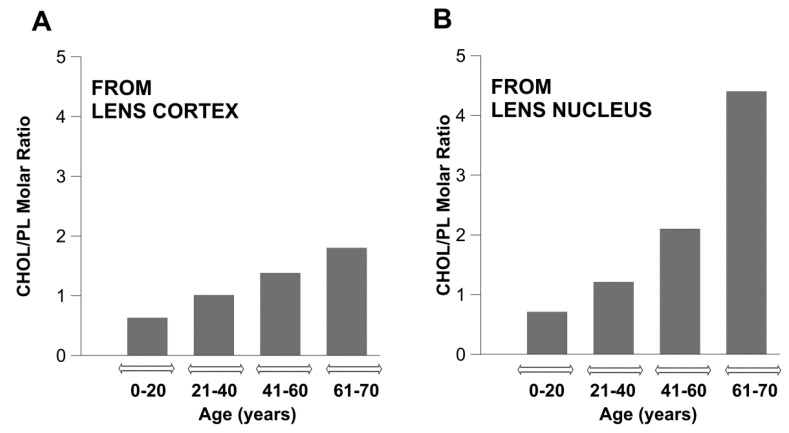
The cholesterol/phospholipid (CHOL/PL) molar ratio in cortical and nuclear lipid membranes of transparent eye lenses from human donors of different age groups. Values taken from [23].

The most unique biochemical characteristic of the fiber cell plasma membrane of the human lens is its extremely high cholesterol content, and that changes drastically with age. In transparent lenses, cholesterol/phospholipid molar ratios are 0.6, 1.0, 1.4, and 1.8 in cortical membranes, and 0.7, 1.2, 2.1, and 4.4 in nuclear membranes, for groups of donors aged 0–20, 21–40, 41–60, and 61–70 years, respectively [23]. As illustrated in Figure 3, the cholesterol content in the nuclear membranes of human lens increases rapidly with age, whereas it increases relatively mildly in cortical membranes. For brevity, these data are presented as the total cholesterol to the total phospholipid isolated from cortical and nuclear fiber cells. However, in the nuclear membranes of persons aged 61–70 years, a portion of the cholesterol forms cholesterol crystals outside of the fiber cell plasma membranes [2]. In this case, the cholesterol content exceeds the cholesterol solubility threshold.

There is no turnover of sterols, lipids, and proteins in old fiber cell membranes, which may function through the repair system during oxidative stress [5,19,24]. Our previous research showed that the high cholesterol content in lens membranes is one of the factors that allows maintenance of the extremely low oxygen concentration in the lens center [2,23]. Due to the age-related changes in cholesterol content and the changes in phospholipid composition, the fiber cell plasma membrane becomes more resistant to oxygen permeation with age, and resistance is greater in the lens nucleus than in the lens cortex [23]. These factors should help to decrease lipid peroxidation and free radical formation in the lens, especially in the lens center, and to maintain lens transparency in old age.

**Figure 4:**
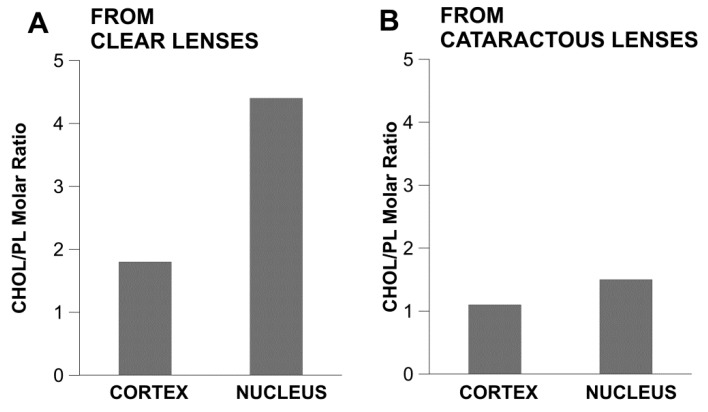
The cholesterol/phospholipid (CHOL/PL) molar ratio in cortical and nuclear lipid membranes of transparent and cataractous eye lenses from 61–70-year-old human donors. Values taken from [2].

As shown in Figure 4, the cholesterol/phospholipid molar ratios for cortical and nuclear membranes isolated from transparent lenses in 61–70-year-old donors are 1.8 and 4.4, respectively. The cholesterol/phospholipid molar ratios for cortical and nuclear membranes isolated from cataractous lenses in the same age group are 1.1 and 1.5, respectively [2]. These results are in agreement with those obtained by Jacob et al. [25] for lipids extracted from whole transparent and cataractous lenses from 73–80-year-old donors showing cholesterol/phospholipid molar ratios of 3.1 and 1.7, respectively. This indicates that the fiber cell plasma membranes of cataractous lenses are characterized by lower cholesterol contents as compared with transparent lenses, and suggests that the high cholesterol content protects lenses against cataract development.

### 2.3. Cholesterol-Lowering Drugs and the Human Diet

Generally, cholesterol is either supplied from the diet (exogenous cholesterol) or synthesized de novo (endogenous cholesterol). Dietary cholesterol accounts for approximately 30% of the total cholesterol in the human body, whereas about 70% of cholesterol is synthesized by the human body [26]. In contrast to other tissues, the uptake of dietary cholesterol by the lens is minimal. Thus, the lens must synthesize, by itself, all the cholesterol needed for fiber cell membrane formation. Some genetic diseases indicate an association between cataracts and defects in the enzymes needed for cholesterol metabolism [27]. One of them, namely Smith–Lemli–Opitz syndrome, is a genetic disease that results from the lack of final cholesterol synthesis and elevated accumulation of 7- and 8-dehydrocholesterol. Children with Smith–Lemli–Opitz syndrome and abnormally low cholesterol levels are mentally deficient and have cataracts [28,29]. The cataract is one phenotype on the clinical spectrum recognized not only for Smith–Lemli–Opitz syndrome but, also, for other genetic diseases with mutation in enzymes of cholesterol synthesis, such as mevalonic aciduria [30,31], cerebrotendinous xanthomatosis [32,33], and X-linked dominant chondrodysplasia punctata [34].

Thus, cataracts are common in genetic disorders with errors in the cholesterol synthesis pathway. It has also been reported that cholesterol-lowering drugs that block the cholesterol synthesis pathway have cataractogenic properties in treated animal groups [35,36,37]. As for the cataractogenic properties of statins in humans, the scientific community is not in agreement. However, it was reported by many authors that blockage of cholesterol biosynthesis by statins leads the development of cataracts [38,39,40,41,42]. On the other hand, Mitchell and Cenedella did not observe any lenticular toxic effects from using lovastatin and simvastatin [43]. Unexpectedly, some authors have observed a decreased risk of cataracts in statin users [44]. Similar to Sant-Gerons et al. [45], we believe that the potential protective effect of statins with regard to cataracts is doubtful and contradicts the need for high cholesterol content in the lens. However, further research is required to solve this problem.

It seems that high dietary cholesterol intake does not affect the cholesterol content in the lens because the lens synthesizes its own lipids, including cholesterol. However, any perturbations that alter cholesterol synthesis may cause unfavorable consequences in the lens lipid membrane structure and disturb lens fiber cell membrane homeostasis.

## 3. Mechanisms Maintaining the Saturating Level of Cholesterol in Phospholipid Bilayers

### 3.1. Lipid Composition Changes

About 5% of human genes are responsible for regulating the lipid composition of cell membranes [6]. Most tissues are sensitive to diet-induced lipid alteration and show high turnover of lipids over days and weeks. By contrast, Nealon et al. provide evidence that the phospholipid composition of the lens is tightly regulated and appears to be independent of diet [15]. In this respect, the lens fiber cell membranes are unique. There is no turnover in the center of the human ocular lens [46] and, as such, oxidation damage accumulates with age. The eye lens adapts to these age-related changes through the lipid modification of fiber cell membranes. The older fiber plasma membranes in the nucleus have higher sphingolipid content and lower glycerophospholipid levels than new fiber cells formed in the cortex. The amount of sphingolipids, including dihydrosphingomyelins and sphingomyelins, increases during aging, in parallel with the decrease in glycerophospholipid (phosphatidylcholine and phosphatidylethanolamine) levels [3,4,5,47,48]. In mature fiber cells, about 2/3 (~66%) of phospholipids are sphingolipids. In young cells, sphingolipids constitute only ~33 mol % of phospholipids. It is well known that sphingolipids (especially dihydrosphingomyelins) are more saturated than glycerophospholipids and exhibit higher resistance to oxidation. Thus, we can conclude that the evolutionarily designed increase in sphingolipid content in fiber cell membranes forms a special mechanism to reinforce the resistance to oxidation in aged membranes. As discussed in Section 2.2, the amount of cholesterol increases as fiber cells mature. The significant increase in cholesterol content in the membrane is accompanied by changes in the lens lipid composition. It is well known that cholesterol solubility is determined by the type of lipid that forms the membrane [49,50]. We were able to determine the cholesterol content at which CBDs and cholesterol crystals start to form in the lipid bilayers—composed of the major phospholipids—of the fiber cell plasma membrane of the human eye lens. The onset of CBD formation in these bilayers was observed at ~33, ~50, ~50, and ~50 mol % of cholesterol in the phosphatidylethanolamine, phosphatidylserine, phosphatidylcholine, and sphingomyelin membranes, respectively [51,52]. At cholesterol contents greater than the cholesterol solubility threshold, the lipid bilayer becomes oversaturated with cholesterol, and cholesterol starts to precipitate in the form of cholesterol crystals. The cholesterol solubility threshold depends on the type of phospholipid forming the bilayer, and was detected at ~50, ~66, ~66, and ~66 mol % in the phosphatidylethanolamine, phosphatidylserine, phosphatidylcholine, and sphingomyelin bilayers, respectively [51,52]. In the fiber cell plasma membranes of the nucleus of transparent lenses of 61–70-year-old human donors, cholesterol has been shown to exist in three different forms: (1) dispersed in phospholipid bilayers as monomers; (2) as aggregates in CBDs; and (3) as crystals [2]. The presented results indicate that in all studied lipid bilayers, the formation of CBDs always precedes the formation of cholesterol crystals, and the appearance of each depends on the type of phospholipid forming the membrane. The high solubility of cholesterol in the sphingomyelin bilayer is associated with forceful interactions between cholesterol molecules and sphingomyelin molecules [13,53]. Interestingly, sphingomyelins are the major phospholipid in human lenses, the content of which increases with age. Sphingomyelin that is in the dihydro form, namely dihydrosphingomyelins, makes up approximately 50% of the phospholipids in the adult lens fiber membranes [4,54]. Thus, changes in the lipid composition are correlated with increased cholesterol levels.

Lens lipid composition also changes dramatically during the development of cataracts. As compared with transparent lenses, the total amount of glycerophospholipids is smaller in cataractous lenses, probably due to their oxidation. Additionally, cataractous lenses contain a greater amount of sphingolipids. Huang et al. demonstrated that sphingolipids increased to 78% of the total phospholipid content in cataractous donors [4].

In transparent lenses, the lipid composition of membranes undergoes significant changes during aging. These lipid age-related changes in the fiber cell membrane help to maintain the transparency of the lens across all ages. However, disturbance of this normal lipid composition through dysfunctions in cholesterol or phospholipid metabolism, or through the oxidation process, may lead to cataract development.

### 3.2. Cholesterol Bilayer Domain Formation

**Figure 5:**
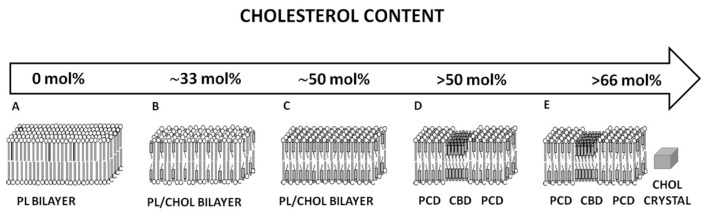
Schematic drawing of changes in the organization of the sphingomyelin bilayer as a function of cholesterol content. (**A**) Pure sphingomyelin bilayer, (**B**,**C**) sphingomyelin/cholesterol bilayer, (**D**) sphingomyelin/cholesterol domain (PCD (phospholipid/cholesterol domain)–bilayer saturated with cholesterol) coexisting with CBD (cholesterol bilayer domain), and (**E**) PCD coexisting with CBDs and new phase cholesterol crystals. Cholesterol limits were taken from [48]. Please note that sphingomyelins account for ~66% of the total phospholipids in the plasma membranes of human eye lens fiber cells [47,54].

When cholesterol content in the sphingomyelin bilayer increases above the upper limit that can be accommodated within the phospholipid bilayer (Figure 5C) (i.e., the cholesterol saturation limit), the excess cholesterol forms CBDs that are supported by the surrounding bilayer (Figure 5D). The next limit, observed at ~66 mol % cholesterol, is the cholesterol solubility threshold, and indicates the total cholesterol content in the phospholipid bilayer that can be dissolved in and supported by the bilayer (in the form of CBDs). Above this limit, a new phase is formed, namely cholesterol crystals (Figure 5E). Cholesterol content in human eye lenses is always high enough to saturate lens fiber cell membranes regardless of the age of the cell [23], due to the presence of CBDs in all human fiber cell membranes, which form the buffering capacity for cholesterol in the surrounding phospholipid bilayer. This is very significant because the saturating cholesterol content in the surrounding bulk phospholipid membranes keeps the physical properties of these membranes consistent and independent of age-related changes in phospholipid composition [23]. The formation of CBDs precedes the formation of cholesterol crystals [51,52].

## 4. Function of Cholesterol in the Lens

### 4.1. Fiber Cell Membrane Homeostasis

**Figure 6:**
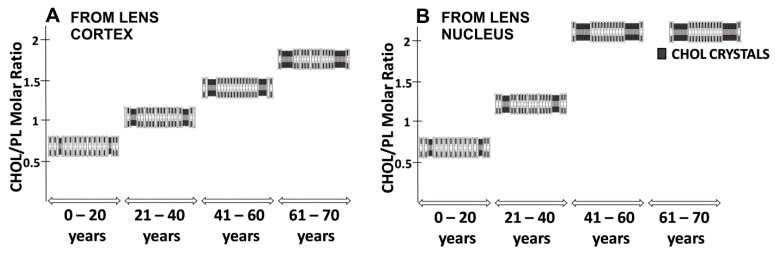
Schematic drawing presenting changes in the organization of cortical (**A**) and nuclear lens (**B**) lipid membranes as functions of age and cholesterol/phospholipid (CHOL/PL) molar ratio in lens membranes. Note that the cholesterol content and size of the CBDs change with age. Also, in the group of 61–70-year-old donors, some of the cholesterol in the nuclear fiber cells formed cholesterol crystals. The shading of domains indicates changes in CBDs size. Adapted from [23].

As indicated previously, the phospholipid composition of the fiber cell plasma membrane changes drastically with age, with increased sphingolipid content and decreased glycerophospholipid content [47,55]. Additionally, phospholipid acyl chain saturation increases with age [18]. The most typical age-related change for fiber cell plasma membranes is increased cholesterol content [56]. High cholesterol content leads to the formation of CBDs within the phospholipid bilayers, as well as to the formation of cholesterol crystals [23,51,52]. These age-related changes in lipid composition should affect membrane properties and organization and make maintaining fiber cell membrane homeostasis difficult, thus altering the lens transparency. We hypothesize that these problems were solved during evolution when mechanisms within the lens were created to ensure a high cholesterol content in fiber cell membranes. Cholesterol plays an important physiological role in the eye lens, and the need for high cholesterol content is validated by the observation that defects in the cholesterol synthesis pathway and the use of cholesterol-lowering drugs contribute to cataract formation (see Section 2.3). Why is high cholesterol beneficial for membrane function? We investigated the bulk membrane properties across membranes formed from lipids extracted from the eyes of human donors of different ages and from different regions of the eye lens [23]. All these membranes had very different phospholipid compositions. However, the cholesterol content was always high enough to saturate the phospholipid bilayer portion of the membranes. CBDs were present in the membranes of donors in all age groups, and cholesterol crystals were detected in the membranes of donors in the oldest age group (i.e., 61–70 years) [23]. Membrane structures are schematically illustrated in Figure 6. Figure 6 presents changes in the organization of cortical and nuclear lens lipid membranes as a function of age and cholesterol content. Cholesterol content changes with age and is different in cortical and nuclear fiber cell membranes. Additionally, the size of the CBDs increases with the age and is always greater in the center of the lens. As discussed in [23], the size of the CBD strongly affects its properties, which also change significantly with age.

Based on our findings, we conclude that the extremely high (saturating) cholesterol content in the fiber cell membrane keeps the bulk physical properties of the lipid bilayer portion of the membrane consistent with, and independent of, changes in the age-related phospholipid composition. Our investigations also allowed us to conclude that the CBDs provide a buffering effect regarding cholesterol concentration in the surrounding phospholipid bilayer, keeping it at a constant saturating level and, thus, keeping the physical properties of the membrane consistent with and independent of changes in phospholipid composition.

### 4.2. Fiber Cell Homeostasis

The lens communication and transport between mature fiber cells is enabled by an extensive network of cell-to-cell junctions (gap and thin junctions) [57] Gap junctions are groups of membrane channels that are permeable to water, ions, and small molecules. Lens gap junctions are built of three types of transmembrane proteins, namely connexins Cx43, Cx46, and Cx50 [58]. Six connexins form a connexon (half-channel). The connection of two connexons from neighboring cells generates an intercellular channel (gap junction). Another transmembrane protein, aquaporin-0 (AQP0), builds water channels. The coupling of two tetramers of AQP0 from neighboring fiber cells creates a thin junction [59].

**Figure 7:**
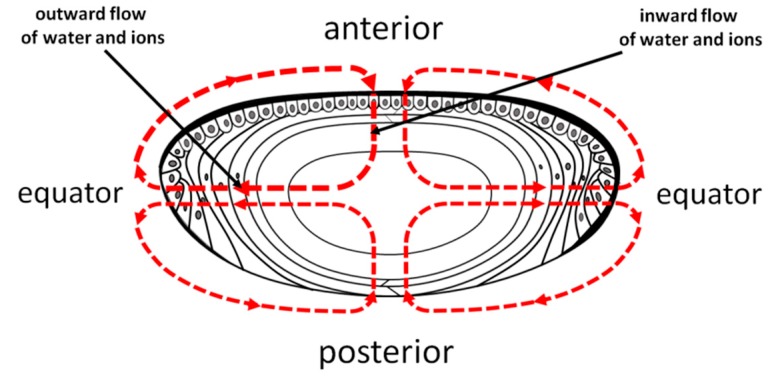
The internal circulatory system of the lens proposed by Donaldson et al. [60].

Based on the lens membrane protein channel network, a specific circulation system, which delivers ions and nutrients deeper into the lens and removes waste products from the central part of the lens, has been proposed by Donaldson et al. [60]. According to Donaldson et al. [60,61], the outward flow of water and ions is located at the equator of the lens where young differentiating fiber cells exist. The inward flow of water and ions is located at the anterior and posterior poles where most of the maturate fiber cells are located (Figure 7).

To ensure that the transport of water and polar molecules between fiber cells is tightly controlled by cell-to-cell junctions, the lipid bilayer portion of fiber cell membranes must provide a high hydrophobic barrier that protects against the uncontrolled leakage of small polar molecules. This condition is fulfilled in fiber cell plasma membranes because of the high, saturating cholesterol content. Human fiber cell plasma membranes are composed of highly saturated phospholipids [18], mainly sphingolipids [4,54]. However, membranes made from saturated phospholipids [62], particularly from saturated sphingomyelins [51], possess very low hydrophobicity of the membrane interior. Only when these membranes are saturated with cholesterol does the hydrophobicity in their centers reach the maximal value, which is comparable to that of hexane [51]. The hydrophobicity profiles across intact fiber cell membranes from lenses of human donors of different age groups were practically identical, independent of the age of the donor and the region of the lens [23]. Their shapes changed from rectangular (observed for model membranes [62]) to bell, and the hydrophobicity in the membrane center was slightly lower than in model membranes. Cholesterol has dramatic effects on the hydrophobicity profiles of phospholipid membranes. The presence of cholesterol (30 mol %/50 mol %) increases the polarity in the polar headgroup region, and significantly decreases the polarity in the central region of the bilayer. At cholesterol contents greater than the cholesterol solubility threshold, the hydrophobicity of the lipid membrane practically does not change. All lens membranes made from lipids isolated from the eye lenses of donors of all ages are saturated with cholesterol. The saturating amount of cholesterol, and not the phospholipid composition, determines the physical membrane properties, including hydrophobicity. Also, the hydrophobicity of the membrane center is lowered by the presence of membrane proteins, although the hydrophobic barrier remains considerably high. Thus, we can conclude that the high (saturating) cholesterol content ensures that the lipid bilayer portion of the fiber cell plasma membrane forms a high hydrophobic barrier for the permeation of polar molecules.

### 4.3. Lens Homeostasis and Maintaining Lens Transparency

The eye lens focuses light on the retina, and must be transparent to perform this function properly. In our opinion, the major mechanism that developed during biological evolution to protect the lens against opacification is the extremely low partial pressure of oxygen within the lens. Any alteration (increase) of oxygen partial pressure within the lens interior, due to long-term hyperbaric oxygen therapy [63,64] vitrectomy surgery, [65], or age [66], almost always leads to cataract formation. Since the lens is avascular, oxygen, like other nutrients, must diffuse to the lens interior. Thus, the three major mechanisms that can control low oxygen partial pressure within the lens are (1) low oxygen partial pressure at the lens surface, (2) oxygen consumption within the lens, and (3) barriers to oxygen permeation created across layers of fiber cells.

At the surface of the anterior and posterior cortex of the transparent healthy lens, the oxygen partial pressure is already as low as 3 mmHg and 9 mmHg, respectively [67]. In the center of the nucleus, oxygen partial pressure reaches a value close to 0 mmHg [68]. Most oxygen (about 90%) is consumed by mitochondria in the outer part of the lens (epithelium and superficial cortex) [68]. Additionally, it is suggested that other systems that depend on nonmitochondrial oxygen consumption exist to remove oxygen from the nucleus. These mechanisms should be formed by ascorbate- [69] and/or glutathione-dependent oxygen consumption reactions [70].

The third mechanism that helps to maintain low oxygen partial pressure within the lens is straightforward, depending on high cholesterol content in the fiber cell plasma membranes. We showed that, because it is saturated with cholesterol, the phospholipid portion (excluding CBDs) of the lens lipid [23] and intact fiber cell membranes [71] possesses a membrane oxygen permeability coefficient significantly lower than the permeation coefficient of a water layer of the same thickness as the membrane. Additionally, in the lenses of older persons, CBDs can occupy a significant portion of the membrane surface, possessing an oxygen permeability coefficient about ten times smaller than that of the bulk phospholipids and significantly increasing the membrane barrier properties [2,72]. Oxygen must pass through thousands of fiber cell plasma membranes when it moves from the lens surface to lens center. Thus, even a very small difference in the oxygen partial pressure created across an individual fiber cell plasma membrane can contribute significantly to the oxygen partial pressure difference between the lens surface and lens center. All these factors indicate that high cholesterol is needed, and even necessary, to protect the lens against oxidative stress.

## 5. Other Tissue/Organ Cells

### 5.1. High Cholesterol and Oxidative Stress

Oxidative stress and high cholesterol levels are major factors leading to the development of atherosclerosis through the inflammatory cascade [73]. In most tissues and organs, including plasma membranes of the smooth muscle cells, the presence of CBDs annunciates the possibility of the appearance of cholesterol crystals, which are a sign of pathological conditions [7]. Interestingly, a large amount of cholesterol and cholesterol crystals in the foam cells of advanced atherosclerotic lesions was found [74]. Thus, there is a clear need for the greater in-depth and specific understanding regarding the roles of cholesterol, CBDs, and cholesterol crystals in the initiation of the atherosclerotic process. The inflammatory nature of atherosclerosis is well established, but the agents that initiate inflammation in the artery wall remain largely unknown. It is suggested that crystalline endogenous substances initiate the inflammation by activating the NLRP3 inflammasome. The NLRP3 receptor can be activated by diverse agents, such as inhaled silica particles and asbestos [75], as well as by urate crystals [76,77,78]. It was assumed that cholesterol crystals also could induce atherosclerosis through activation of the inflammasome pathway. The finding that the deposition of minute cholesterol crystals in arteries is an early cause—rather than a late consequence—of inflammation provides new insight into the pathogenesis of atherosclerosis [79]. This discovery suggests that research aimed at the formation of minute cholesterol crystals may contribute significantly to an understanding of how these crystals are related to the inflammatory process leading to the development of atherosclerosis. Two pathways may be involved in the formation of intracellular minute cholesterol crystals. First, minute cholesterol crystals may be formed through the uptake of the oxidized low-density lipoproteins (LDL) and the release of free cholesterol. Secondly, cholesterol crystals may be formed through the peroxidation of membrane phospholipids, the formation of CBDs, and their dissolution into minute cholesterol crystals [80]. Independent of either pathway, minute cholesterol crystals can activate inflammasomes and induce inflammation, which may lead to the development of atherosclerosis. In the first pathway, a key role is played by oxidized LDL [81,82]; in the second pathway, the key role is assigned to membrane CBDs, which are precursors to cholesterol crystals [52]. Previously, macroscopic cholesterol crystals were assumed to be a late consequence of inflammation, rather than being an initiator [83,84]. After macroscopic cholesterol crystals are formed, they cause mechanical damage through physical injury to cells and plaque rupture, which may trigger local and systemic inflammation [85,86,87].

It is well known that several physical factors, such as saturation, temperature, pH, and hydration, trigger cholesterol crystallization [88]. Additionally, the occurrence of cholesterol crystals may be enhanced by the effects of lipid peroxidation [89,90]. Phospholipid acyl chain unsaturation drastically decreases the cholesterol solubility threshold in model membranes as well as the cholesterol concentration at which CBDs start to form [91,92,93]. If we compared the unsaturated 1-palmitoyl-2-arachidonoyl-*sn*-phosphatidylcholine lipid, which forms a bulk bilayer, with a saturated membrane composed of sphingomyelin, we may observe an earlier occurrence of CBDs at ~33 mol % and cholesterol crystals at ~50 mol % (for sphingomyelin, CBDs start to from at ~50 mol % and cholesterol crystals appear at ~66 mol %) [86]. Both our own observations [86] and those of other studies [92,93] indicate that the introduction of polyunsaturated acyl chains in lipid membrane, as well as the peroxidation of these chains, decreases the cholesterol content at which CBDs and cholesterol crystals start to form.

### 5.2. Cholesterol-Lowering Drugs and the Human Diet

The liver plays a central role in the metabolism and regulation of the cholesterol level in the human body [94]. All cells also develop their own machinery for the synthesis of cholesterol [94]. In contrast to tissues with low phospholipid turnover, such as the lens [46] and brain [95], the cells of most tissues absorb small amounts of cholesterol from the dietary source. Cholesterol in the avascular lens and in the brain, isolated by the blood–brain barrier, is synthesized in situ. Similar to the lack of clarity about the effect of statins on cataract development, our understanding of the effect of statins on cognition and brain function is incomplete. On the one hand, statins have been associated with cognitive impairment [96,97]. On the other hand, there are publications that provide evidence opposing the association of statins with cognitive impairment [98,99]. Additionally, some epidemiologic studies report a lower risk of dementia in statin users [100,101], or even that statins provide a protective effect against dementia and Alzheimer’s disease [102]. In some epidemiologic studies, a beneficial effect was not found [103,104]. It must be noted that differences in data and conclusions may arise depending on the transport mechanisms studied. Generally, hydrophobic statins are known to cross the blood–brain barrier, whereas hydrophilic statins are not thought to cross the barrier. Most of the cholesterol in the brain is present in myelin sheaths, however, neurons also contain large amounts of cholesterol. Cholesterol synthesis and utilization differ among the different types of brain cells. Thus, pharmacological manipulation might produce different responses in the various types of the brain cells. In their review, Schultz et al. [105] explain why it is possible for cholesterol-lowering drugs to provide both detrimental and protective effects on brain function. The authors associate cognitive impairment in statin users with low cholesterol content in cells, which may lead to higher fluidity of neuronal membranes and, thereby, affect neurotransmission. With regard to the use of statins to treat dementia, the authors propose the beneficial mechanisms of cholesterol synthesis inhibitors on the cerebrovascular system. Thus, their hypothesis [105] suggests that, similar to cardiovascular diseases, high cholesterol content is deleterious to the vascular system of the brain, whereas similar to the lens, high cholesterol content is beneficial for the myelin membrane. The effect of cholesterol on brain tissue is dual. To perform an especially rapid propagation of electrical impulses (called saltatory conduction), nerve axons must be electrically isolated from the environment. The saturating cholesterol content (which has not yet reached the level needed for CBD formation) serves this function well. This high cholesterol content ensures the highest hydrophobicity of the membrane. The additional enhancement of the insulating properties of these membranes is due to a high sphingolipid content. These physicochemical properties, which are ensured by the high cholesterol content, are crucial for myelin membranes. These membranes do not contain enough proteins (15–30% by weight) to overcome nonspecific leakage through the lipid bilayer portion of the membrane. However, a high cholesterol level in the brain can damage the cerebrovascular system. To better understand the effects of high and low cholesterol contents in biological membranes, we direct readers to the review [106].

A close link between elevated plasma cholesterol (especially a high LDL content in the blood) and atherosclerosis has been known for many years. It was assumed that the reduction of plasma cholesterol content by statins would be associated with a reduced risk for atherosclerosis. Patients with familial hypercholesterolemia make up 0.2% of the population [107] and usually have around 10 times higher total serum cholesterol content than healthy people [108]. This draws attention to the role of high cholesterol in the progression of atherosclerosis. However, in people without familial hypercholesterolemia but with a high LDL level, the oxidation of these lipoproteins induces inflammation rather than high cholesterol content [109]. High cholesterol level contributes to atherosclerosis progression only in an indirect and complex manner. In combination, high cholesterol content, oxidative stress, oxidized LDL, and autophagic machinery contribute to the development of atherosclerosis. Some authors have shown that CBDs can spontaneously transform into cholesterol crystals [110,111,112]. These results demonstrate that when the lipid bilayer is saturated by cholesterol and contains CBDs, it can be the nucleation site of cholesterol crystals. This supports the hypothesis that cell membranes may induce nucleation of extracellular crystals in the early stages of atherosclerosis [111,113]. We [23,52] and other authors [111] have previously shown, in model membranes, that CBDs can be nucleating sites for the formation of cholesterol crystals. Additionally, immiscible CBDs were observed in arterial smooth muscle membranes [114], and cholesterol crystallization from membranes of model macrophage foam cells was identified [115].

## 6. Conclusions

The terms “good cholesterol” and “bad cholesterol” are overused in scientific and popular literature. Therefore, in our review, we focus on the beneficial and negative actions of cholesterol; in particular, we focus on high cholesterol, which functions differently in the eye lens than in other tissues and organs. The major difference between cholesterol action in the lens versus in other tissues and organs is that the eye lens is avascular, and other human body tissues and organs are exposed to blood and its related components, including cholesterol transported in LDL and HDL, oxygen transported by red blood cells, and all cells of the immune system. Additionally, differentiation of lens fiber cells involves formation of an organelle-free zone comprising cells devoid of organelles. Since the organelle-free zone in the lens consists of only the plasma membranes and cytosol, the high cholesterol content seems to be important and beneficial.

For the elderly human population, the cholesterol content in human eye fiber cell plasma membranes is often high enough to induce formation of cholesterol crystals, presumably outside the fiber cell membranes [2]. Most likely, CBDs form the precursors for minute cholesterol crystals [2,52]. Do these crystals harm the lens in the same way as they harm other tissues and organs? As we indicate in this review, high cholesterol content and the presence of CBDs in fiber cell plasma membranes are beneficial to the eye lens. However, we cannot confirm that the formation of cholesterol crystals is beneficial to the eye lens. Our results indicate that formation of minute cholesterol crystals, through the fiber cell plasma membrane pathway, is not harmful for aged human lenses, where cholesterol crystals have already been detected [2]. This is because inflammation does not appear to play a role in cataract formation. The lens fiber cells lose their organelles soon after they are formed [116,117]; this protects them from the harmful induction of inflammasomes by minute cholesterol crystals. Also, lens fiber cells are protected from initiation of the inflammatory cascade because the lens is avascular. Thus, cholesterol crystals can be formed without harmful effects on lens properties, such as transparency, and lens functions.

In our opinion, the second major factor that distinguishes between the beneficial action of cholesterol in eye lens and its negative action in other tissues and organs is the difference in exposure to molecular oxygen. What can exposure to molecular oxygen do in terms of cholesterol action? It can induce lipid oxidation, especially in tissues and organs with membranes made of highly unsaturated phospholipids. This process has a straightforward connection with the formation of CBDs and cholesterol crystals in these membranes. It was shown that phospholipid unsaturation [92,93] and the formation of phospholipid peroxides [89,91] significantly decreases the threshold of cholesterol content in the membrane at which CBDs and cholesterol crystals start to form. Thus, even in the plasma membranes of typical cells (not eye lens fiber cells), severe (acute) oxidative stress can induce the formation of minute cholesterol crystals from plasma membranes, as suggested in [7,80,89]. Since all cell organelles are present in typical cells, and because cells have contact with interstitial fluid, the inflammatory cascade can be initiated, indicating a negative pathological action of cholesterol.

The opposite situation exists for eye lens fiber cell plasma membranes. At first, these membranes are built from highly saturated phospholipids. Additionally, oxygen concentration in the lens, especially in the lens center, is very low. Thus, lipid oxidation cannot accelerate formation of cholesterol crystals; they can be formed only when plasma membranes have a very high cholesterol content, which is only reached in old age. Additionally, the lack of cellular organelles and the lack of contact with blood protects fiber cells from the negative action of cholesterol via the inflammatory cascade.
